# Staged management of giant bilateral perinephric adipocytic neoplasms

**DOI:** 10.1002/ccr3.828

**Published:** 2017-03-23

**Authors:** Aileen Grace P. Arriola, Edmund K. Bartlett, Paul J. Zhang, Kumarasen Cooper, Ali Naji, Robert E. Roses

**Affiliations:** ^1^ Pathology and Laboratory MedicineHospital of the University of PennsylvaniaPhiladelphiaPennsylvaniaUSA; ^2^ Department of SurgeryHospital of the University of PennsylvaniaPhiladelphiaPennsylvaniaUSA; ^3^ Endocrine and Oncologic SurgeryHospital of the University of PennsylvaniaPhiladelphiaPennsylvaniaUSA

**Keywords:** Liposarcoma, perinephric mass, retroperitoneal lipoma

## Abstract

We present a patient with giant bilateral perinephric masses favored to represent liposarcoma preoperatively. Bilateral renal involvement posed a clinical challenge; careful histologic assessment and surgical planning allowed preservation of renal function.

## Case Presentation

A 52‐year‐old Caucasian man with multiple sclerosis presented in August 2014 for worsening chronic abdominal distension with associated lower extremity edema. These symptoms began in 2010 at which time radiologic work‐up revealed bilateral, large retroperitoneal masses. An initial biopsy of these masses was reportedly benign, and hence, the patient did not pursue further intervention. Over time, he developed complications related to progressive enlargement of the lesions, including multiple hospital visits for lower extremity cellulitis in the setting of chronic venous stasis ulcers. In 2014, he presented to our institution with progressive abdominal distension with associated severe dyspnea and limited mobility (only 10 steps with the assistance of a walker).

**Figure 1:**
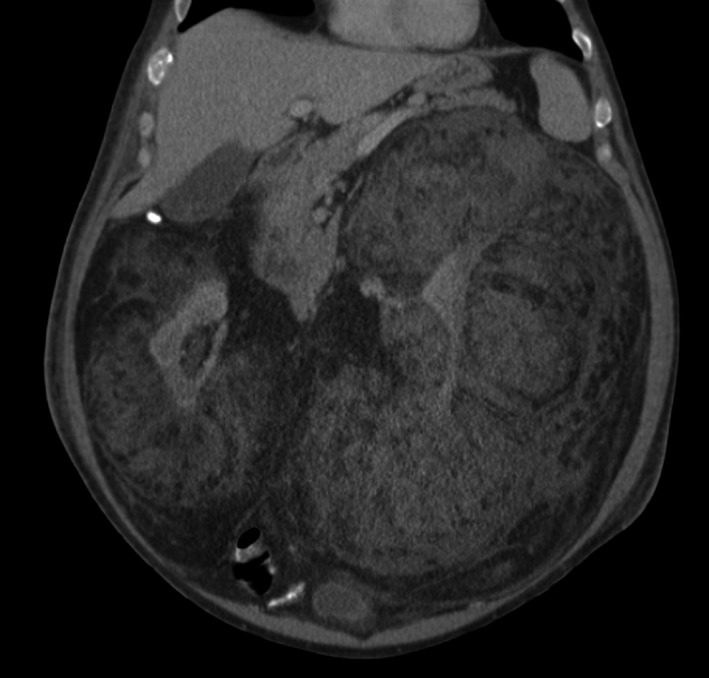
Massive bilateral perinephric masses with near‐complete obliteration of the left kidney and encasement of the right kidney.

A CT of the abdomen and pelvis revealed massive bilateral, well‐encapsulated, fatty retroperitoneal masses encasing both kidneys, compressing the vena cava, and measuring 38.1 × 24.4 × 18.6 cm on the left and 35.7 × 23.5 × 14 cm on the right (Fig. 1). The radiologic findings suggested a diagnosis of retroperitoneal liposarcoma. Renal angiomyolipoma, ganglioneuroma, schwannoma, lymphoma, and renal cell carcinoma were also included in the differential. A serum IgG4 level was within normal limits. He had normal renal function and renal scintigraphy revealed 70% of function in the right kidney. Additional imaging did not reveal metastatic disease.

A staged approach was planned in an effort to preserve kidney function while obtaining definitive pathology and minimizing operative morbidity. The first operation was an *en bloc* resection of the mass on the left with the left kidney, preserving the left adrenal gland. The patient recovered well from this operation.

## Pathologic Evaluation

### Gross findings

**Figure 2:**
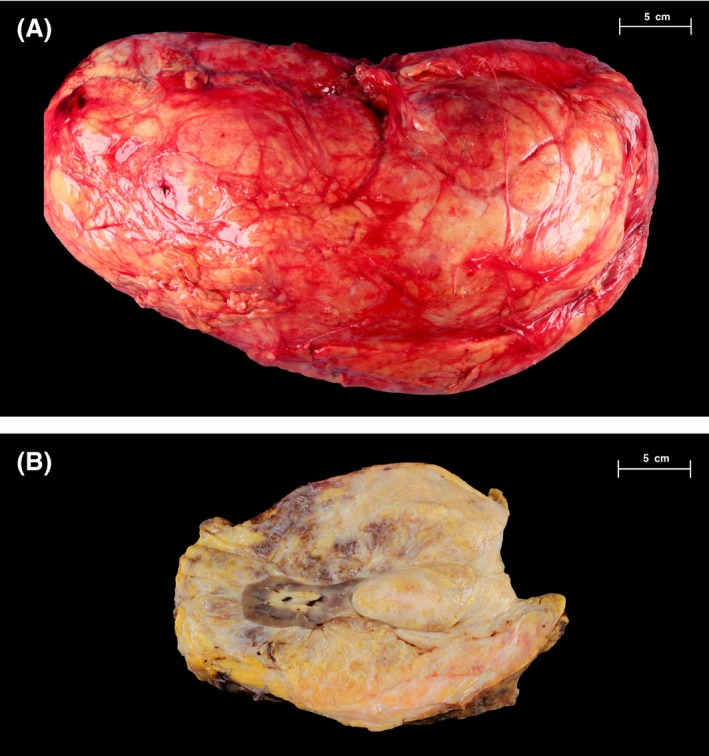
(A) Left perinephric mass resected *en bloc* with left kidney, (B) depicts the cut surface of the mass.

The left retroperitoneal mass was 47 cm in greatest dimension and weighed 12.5 kg with a well‐encapsulated, firm outer surface (Fig. 2). Serial sectioning revealed a marbled pale‐yellow to tan fatty cut surface with dissecting white fibrotic bands. The mass was adherent to the kidney capsule, with no infiltration into the parenchyma.

### Histopathology

**Figure 3:**
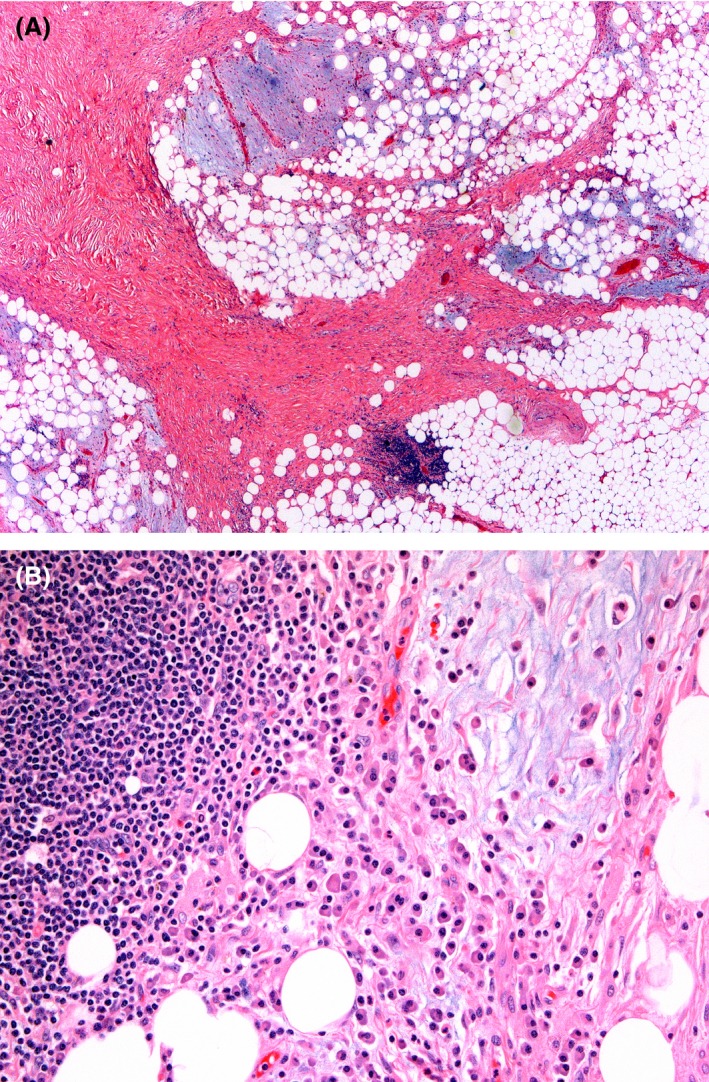
Histology reveals benign adipocytic and fibrous components with a myxoid background containing plasma cells and lymphocytes. (A‐25x, B‐200x).

The lesion consisted of mature fat entrapped by a fibrous proliferation, myxoid background, and a noticeable lymphoplasmacytic infiltrate. There was only focal variation in the size of adipocytes and no atypical or hyperchromatic adipocytic or stromal nuclei were seen on extensive sampling (Fig. 3). An IgG4 immunostain highlighted increased IgG4 plasma cells (>10% of all plasma cells). Fluorescence in situ hybridization (FISH) for MDM2 and CDK4 amplification was negative. FISH for ALK translocation was also performed, which was negative.

### Differential diagnosis

The differential diagnoses based on the histomorphology included retroperitoneal well‐differentiated sclerosing/inflammatory liposarcoma, dedifferentiated liposarcoma, IgG4‐related sclerosing disease, and retroperitoneal lipoma.

Based on the location and massive size of the lesion, a liposarcoma (either well‐differentiated or dedifferentiated) would be the most frequent diagnosis. A dedifferentiated liposarcoma with inflammatory myofibroblastic tumor‐like features is a variant wherein the dedifferentiated component is virtually identical to an inflammatory myofibroblastic tumor 1. Our case was more consistent with this entity due to the prominent fibrous bands with inflammatory cells. However, MDM2/CDK4 amplification is a distinctive molecular event identified in almost all well‐differentiated and dedifferentiated liposarcomas. The negative FISH studies and lack of atypical nuclei made this diagnosis unlikely.

The increase in IgG4 plasma cells raised the possibility of an IgG4‐related sclerosing process. On histologic grounds, such diseases are characterized by both an increase in IgG4 plasma cells, storiform fibrosis, and obliterative phlebitis 2. Although our case contained increased IgG4 plasma cells, this finding can be ubiquitous in chronic inflammatory conditions 3. Our case lacked obliterative fibrosis, and importantly, no encasement of retroperitoneal vessels was noted on radiology. Hence, an IgG4‐related disease was considered less likely. Moreover, the serum IgG4 level was normal.

Retroperitoneal lipomas are an extremely rare finding and only diagnosed after thoroughly excluding liposarcoma on morphologic and molecular genetic grounds. There have been at least 40 cases in the English literature; some presented with giant abdominal tumors 4, 5, 6, 7, 8, 9, 10, 11, 12, 13, 14, 15, 16, 17, 18, 19, 20, 21, 22. The majority of the reports describe typical lipomas with pure adipocytes on histology. There are two reports that describe retroperitoneal fibrolipomas; neither report included results of molecular studies and both patients were free of disease at 12 months and 5 years of follow‐up 13, 14.

Due to the presentation of bilateral massive perinephric masses with a bland adipocytic component, the lesion warranted a diagnosis of a well‐differentiated adipocytic neoplasm. This diagnosis conveys that the lesion is not malignant but also implies the possibility of local recurrence.

## Outcome and Follow‐up

**Figure 4:**
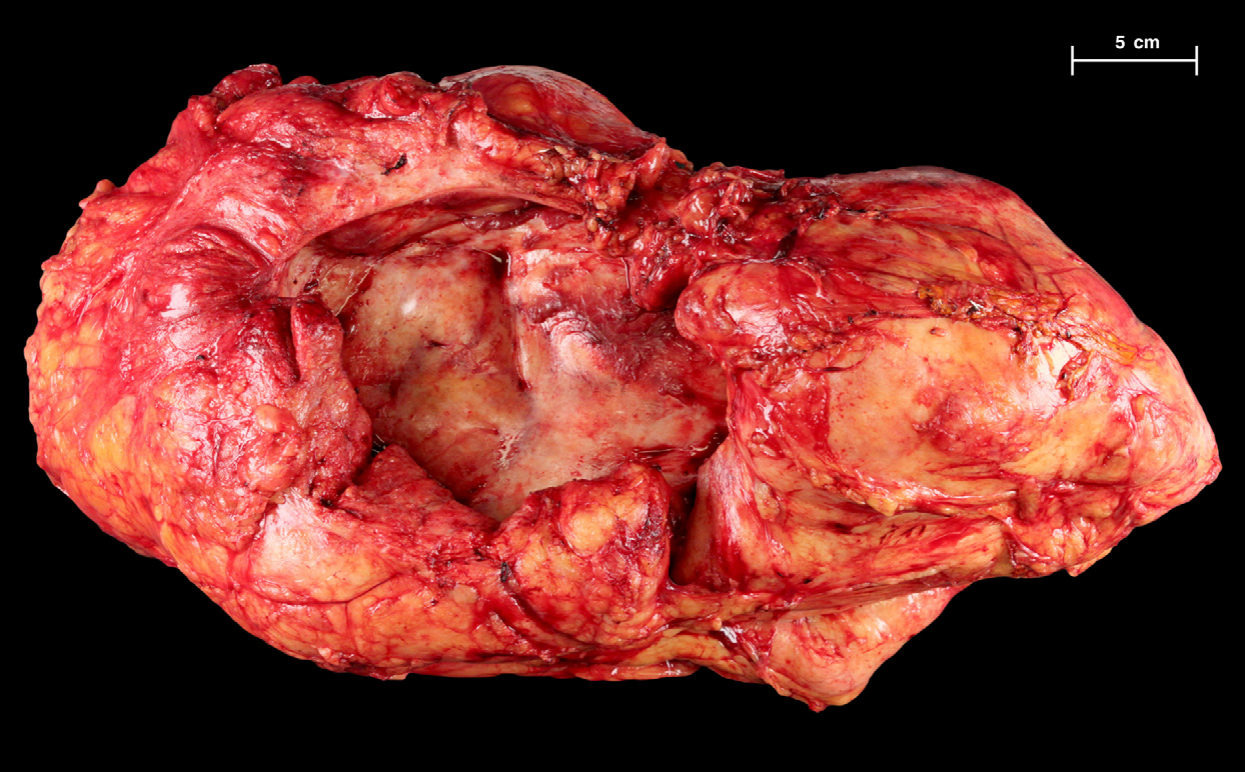
Resected right perinephric mass. Mass encased but did not invade the kidney, and thus, kidney was able to be preserved.

**Figure 5:**
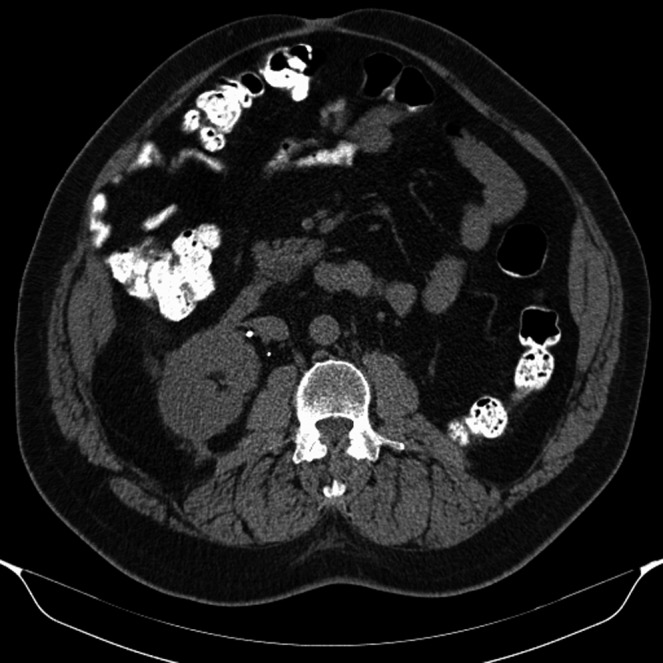
CT scan obtained 1 year after second resection. No evidence of recurrent disease. The right kidney has returned to its natural morphology and kidney function remains adequate.

Given the benign pathology of the left‐sided mass, the right perinephric mass was not resected initially. Six months after his first resection, the patient reported recurrent symptoms of lower extremity edema and dyspnea. Repeat CT scan at that time revealed mild progression of the right‐sided mass with worsening hydronephrosis. The patient was scheduled for resection with the intent of kidney preservation or autotransplantation, if the kidney could not be safely dissected from the mass in situ. At operation, the kidney was successfully mobilized from the mass, which was removed intact (Fig. 4). Final pathology was consistent with that of the original mass. The patient continues to do well and was disease free at the time of last follow‐up, 1 year after his second operation (Fig. 5).

## Conflict of Interest

None declared.

## Authorship

AGPA: involved in manuscript write‐up (key clinical message and pathology) and contributed gross photographs and microscopic pictures; EKB: involved in manuscript write‐up (clinical information and follow‐up) and contributed radiologic images; PJZ and KC: performed manuscript review, differential diagnosis, revisions, and editing; AN and RER: performed manuscript review and editing.
